# Integration of Hydrogel Microparticles With Three-Dimensional Liver Progenitor Cell Spheroids

**DOI:** 10.3389/fbioe.2020.00792

**Published:** 2020-07-21

**Authors:** Stefan D. Gentile, Andreas P. Kourouklis, Hyeon Ryoo, Gregory H. Underhill

**Affiliations:** Department of Bioengineering, University of Illinois at Urbana-Champaign, Champaign, IL, United States

**Keywords:** tissue engineering, microenvironment, liver, spheroid, microparticles, polyethylene glycol (PEG)

## Abstract

The study of the liver progenitor cell microenvironment has demonstrated the important roles of both biochemical and biomechanical signals in regulating the progenitor cell functions that underlie liver morphogenesis and regeneration. While controllable two-dimensional *in vitro* culture systems have provided key insights into the effects of growth factors and extracellular matrix composition and mechanics on liver differentiation, it remains unclear how microenvironmental signals may differentially affect liver progenitor cell responses in a three-dimensional (3D) culture context. In addition, there have only been limited efforts to engineer 3D culture models of liver progenitor cells through the tunable presentation of microenvironmental stimuli. We present an *in vitro* model of 3D liver progenitor spheroidal cultures with integrated polyethylene glycol hydrogel microparticles for the internal presentation of modular microenvironmental cues and the examination of the combinatorial effects with an exogenous soluble factor. In particular, treatment with the growth factor TGFβ1 directs differentiation of the spheroidal liver progenitor cells toward a biliary phenotype, a behavior which is further enhanced in the presence of hydrogel microparticles. We further demonstrate that surface modification of the hydrogel microparticles with heparin influences the behavior of liver progenitor cells toward biliary differentiation. Taken together, this liver progenitor cell culture system represents an approach for controlling the presentation of microenvironmental cues internalized within 3D spheroidal aggregate cultures. Overall, this strategy could be applied toward the engineering of instructive microenvironments that control stem and progenitor cell differentiation within a 3D context for studies in tissue engineering, drug testing, and cellular metabolism.

## Introduction

During liver development, liver progenitor cells, termed hepatoblasts, differentiate into hepatocytes, which comprise the majority of the liver tissue and biliary epithelial cells (cholangiocytes) that line the bile ducts (Strick-Marchand and Weiss, 2002; Strick-Marchand et al., 2004). These bipotential liver progenitor cells play an important role in adult liver regeneration and the progression of liver diseases including fibrosis (Hanley et al., 2008; Carpentier et al., 2011). The complex architecture of the liver microenvironment exposes these cells to various physical and chemical stimuli that can influence their differentiation trajectories. It has been shown that the growth factor TGFβ1 can push these progenitor cells toward the cholangiocytic fate and that substrate stiffness and other biochemical signals can further influence liver progenitor differentiation (Clotman et al., 2005; Lemaigre and Clotman, 2005; Gyorfi et al., 2018). To date, many techniques have been used to approximate the liver microenvironment. Recently, there has been extensive work with cellular microarrays to create a high-throughput system of microenvironments independently tunable for extracellular matrix proteins linked to other chemical signals (Kaylan et al., 2016). This can also be paired with substrates of various stiffnesses to investigate combinatorial physical and chemical effects (Kourouklis et al., 2016).

Much of the previous work dissecting the liver microenvironment has been done in two-dimensional (2D) systems that allow for a great degree of control. However, utilizing a three-dimensional (3D) culture configuration allows for more complexity and *in vivo-*like conditions (Legant et al., 2009; Underhill et al., 2012). Namely, this allows for differential cell–cell interactions, cellular organization, and environmental cues that may be missing in a simple 2D monolayer system. Toward the development of suitable 3D culture models, one approach consists of cells encapsulated within a hydrogel in order to control for aggregate shape, size, and surrounding chemistry. Cells can also be forced to self-aggregate into spheroids ranging in size from a few cells to millions (Goude et al., 2014).

Three-dimensional spheroids can be further modified by the addition of artificial micro- or nanoparticles to control the internal structure, act as carriers for various factors, or to present different functional groups to the aggregating cells (Bratt-Leal et al., 2011). Previous experiments have used a wide variety of materials to construct microparticles, including simple metallic and ceramic microparticles (Palombella et al., 2017), mineral coated plastics (Khalil et al., 2017), and particles manufactured with agarose, gelatin (Fan et al., 2008; Bratt-Leal et al., 2011; Tabata and Tajima, 2017), or extracellular matrix (ECM) proteins such as collagen. Polymers such as poly(lactic-co-glycolic acid) (PLGA; Solorio et al., 2010; Bratt-Leal et al., 2011) or polyethylene glycol (PEG; Ravindran et al., 2011; Parlato et al., 2013) have been used to create hydrogel microparticles via photo- or chemical crosslinking (Parlato et al., 2013; Ahmed, 2015). These particles were further modified to express surface ligands or proteins to present different chemical signals.

The method of microparticle-imbued spheroids has been used extensively to study tumor formation and to model *in vivo* cancer conditions (Weiswald et al., 2015; Song et al., 2016; Ishiguro et al., 2017; Ferreira et al., 2018). Three-dimensional spheroids have also advanced the study of stem cell microenvironments (Bratt-Leal et al., 2009; Rivron et al., 2018). Poly(lactic-co-glycolic acid), PEG, and hyaluronic acid based microparticles have all been used to induce or modulate differentiation (Bratt-Leal et al., 2011; Ravindran et al., 2011; Ansboro et al., 2014). It has been shown that simply the physical presence of microparticles within a pluripotent stem cell aggregate can change the differentiation phenotype (Bratt-Leal et al., 2011; Baraniak et al., 2012). Coupling the physical effects of microparticles with growth factors in human mesenchymal stem cell spheroids can tune chondrogenesis (Ravindran et al., 2011; Ansboro et al., 2014; Goude et al., 2014). Further surface functionalization of the hydrogel can also be used to sequester proteins within the spheroid (Rinker et al., 2018).

In this report, we demonstrate an approach to integrate PEG microparticles into liver progenitor spheroids to create 3D models of liver microtissues with controllable physical and biochemical cues. In the absence of a supporting scaffold, we incorporated hydrogel particles in liver progenitor spheroids, with control over their presentation density and surface chemistry. Despite the lack of control over the particles position within the spheroids, our studies revealed the combinatorial effects of TGFβ1 and hydrogel particles on cell behavior. In summary, our studies showed that the addition of a sufficient number of particles among the liver progenitor cells during spheroidal aggregation leads to an enhancement in biliary differentiation. Specifically, we demonstrate that the combined presentation of hydrogel particles and TGFβ1 significantly increased the expression of biliary markers. Further, we found that the surface modification of the hydrogel particles with heparin and their subsequent incorporation in 3D spheroids provided another route to control the extent of biliary differentiation in the presence of TGFβ1.

## Materials and Methods

### Formulation of Microscale PEG Hydrogels

Biotinylated PEG hydrogel microparticles were fabricated through acrylamide crosslinking between PEGDA (3.4 kDa, Laysan Bio, ACRL-PEG-ACRL-3400-1GR) and Acry-PEG-biotin (5 kDa, Nanocs, Cat.#: PG2-ARBN-5k). The reaction performed within an emulsified mixture of water-separated PEG and Dextran phases that are rich in their corresponding components. Specifically, 1 volume part of PEGDA (24%w/v) was mixed with 7.2 parts of Dextran (40%w/v, 40 kDa, Sigma Aldrich, 31389), 4.8 parts of magnesium sulfate anhydrous reagent (40%w/v, SCS Storeroom, 34533000) and 1.4 parts of Acrylate-PEG-Biotin (3.5%w/v). Irgacure 2959 (0.25%w/v, BASF, 55047962) was added to the polymer mixture with a volume percentage of 10%v/v volume. All the components were dissolved in PBS (pH 8) with 0.22 M potassium chloride and were subject to vortex for 1min following their initial mixing. The resulted emulsion was allowed to equilibrate for 20min before it became subject to UV light via an OmniCure S1500 Spot UV Curing System (Excelitas Technologies) with Fiber Light Guide (320–309 nm filter) at 13% (approx. 560 mW/cm^2^). The crosslinked particle suspension was diluted 40 times in dH_2_O and placed for centrifugation at 4,000 × *g* for 4 min. Subsequently, the supernatant was exchanged with dH_2_O (2×) to complete particles cleaning. The average size of the formed hydrogels was monitored through bright field microscopy.

Toward heparin presentation, particles were incubated with streptavidin (50 ug/ml, VWR, 97062-810) and trace amounts of Alexa Fluor 647-conjugated streptavidin (Invitrogen, Cat.#: S-21374) to support fluorescent microscopy studies. Following streptavidin conjugation on the microscopic hydrogels, the latter were further incubated with biotin-heparin (1 mg/ml, Sigma Aldrich, B9806-10MG). The various hydrogel particles were rinsed through a 5 μm cut-off filter (PluriSelect, 43-50005) combined with further PBS rinsing. The collected hydrogels were counted and added to cell suspensions at selected cells to particle ratios.

### Formation of Liver Progenitor Cell Spheroids

We utilized bipotential mouse embryonic liver (BMEL) 9A1 cells between passages 30 and 33 that were cultured as previously described (Strick-Marchand and Weiss, 2002). Collagen I (0.5 mg/ml) solution was prepared and used to coat a T-150 tissue culture plastic flask over before seeding cells under controlled environmental conditions (37°C and 5% CO_2_). Cells were treated with trypsin-EDTA (0.25% [v/v] for ≤10 min) and then subjected to centrifugation (800 × *g* for 5 min) for pellet formation and counting. Basal media for expansion consisted of Roswell Park Memorial Institute (RPMI) 1640 + Glutamine (Life Technologies, 61870-127) and fetal bovine serum (10% [v/v], FBS), penicillin/streptomycin (1% [v/v], P/S), human recombinant insulin (10 μg/ml, Life Technologies, 12585-014), IGF-2 (30 ng/ml, PeproTech, 100-12), and epidermal growth factor (EGF) (50 ng/ml, PeproTech, AF-100-15). Differentiation media consisted of Advanced RPMI 1640 (Life Technologies, 12633-012) with FBS (2% [v/v]), P/S (0.5% [v/v]), L-glutamine (1% [v/v]), and minimum non-essential amino acids (1% [v/v], Life Technologies, 11140-050). For spheroid culture, the cells were seeded in 96-well ultra-low-attachment (ULA) plates at a density of 20E3 cells/well and with the corresponding differentiation media. Smaller spheroids were created using AggreWell^TM^400 plates (Stemcell Technologies, 24 well variant, 34411). The cell mixtures were added to the plates at a seeding density such that the individual spheroids would initially have 1E3 cells/spheroid. In this process, cells were concentrated at 6E5 cells/ml of growth media and 2 ml of cell suspension was added to each well of the AggreWell plate (1.2E6 cells/well). Using a balance plate, the AggreWell plate was spun at 100 × *g* for 3 min, forcing cells into the smaller microwells that were incorporated in the bottom plate to form cell aggregates.

### Co-culture of Liver Progenitor Cell Spheroids With Microscale Hydrogels

For the fabrication of cell spheroids with microscopic hydrogels, the latter were mixed with BMEL 9A1 cells at certain cell-to-particle ratio (no particles, 2:1 5:1, 20:1, and 100:1) and then applied within the corresponding 96-well ULA plates or AggreWell^TM^400 plates. During all the different conditions of cell-particle co-culture, 20E3 cells/well was used as seeding density in ULA plates and 1E3 cells/microwell was used in the AggreWell plates, unless otherwise specified.

### Immunostaining of Spheroid Cultures

Cell spheroids were treated with brefeldin A (10 μg/ml, R&D Systems, 1231/5), an inhibitor of protein translocation to the Golgi that facilitates intracellular immunostaining of secreted factors such as OPN, for 2 h and subsequently fixed in paraformaldehyde (4% [v/v] in 1 × PBS) for 1 h. Fixed samples were permeabilized with Triton X-100 (1% [v/v] in 1 × PBS) for overnight incubation under 4°C and then washed with PBS three times for 5 min each before they got incubated in blocking buffer (5% [v/v] donkey serum in 1 × PBS, with 0.25% [v/v] Triton X-100) for 1 h at room temperature. We incubated samples for 24 h at 4°C and with continuous rocking with one or more of the following primary antibodies diluted in blocking buffer: mouse anti-ALB (1/50 from stock, R&D Systems, MAB 1455) and goat anti-OPN (1/60 from stock, R&D Systems, AF808). We next incubated samples for 24 h at room temperature with one or more of the following secondary antibodies diluted in blocking buffer: DyLight 550-conjugated donkey anti-mouse IgG (1/50 from stock, Abcam, ab98767) and DyLight 488-conjugated donkey anti-goat IgG (1/50 from stock, Abcam, ab96935). Samples were immersed in DAPI solution for 24 h at 4°C and then mounted in Fluoromount G with DAPI (Southern Biotech, 0100-20) and imaged the next day using a Zeiss LSM 700 confocal microscope (Carl Zeiss, Inc.) and associated Zen Pro software. In order to stain the smaller, AggreWell spheroids, the staining times were reduced to 25% to avoid oversaturation and nonspecific attachment. In order to capture entire spheroid volumes, we got sections of cell spheroid for determined intervals of 1–5 μm in the *z*-axis. Intensity was measured in ImageJ with at least 3 spheroids imaged for each condition with at least three biological replicates. Data was represented as a mean ± SEM, unless otherwise noted.

### RNA Isolation and qRT-PCR Analysis

Trizol solution (Life Technologies 15596-026) was used to collect RNA from cell spheroids that were cultured for the referred periods. For larger, ULA spheroids, 16–24 spheroids were pooled for RNA collection, while several hundred of the smaller, AggreWell-formed spheroids were used. The collected RNA was later isolated through phenol-chloroform extraction following manufacturer’s instructions. Samples were digested through DNAse (New England Biolabs, M0303S) at 37°C for 30 min and cleaned using an RNeasy Mini Kit (Qiagen, 74104). RNA concentration was measured by UV spectroscopy and only samples with a ratio >1.8 were used for cDNA preparation. cDNA from isolated RNA was generated using the Supermix iScript cDNA synthesis kit (BioRad, 1708841) and mixed with pre-added primer pairs at a final concentration of 100 nM/primer, again per manufacturer’s instructions. Primer pairs for each gene were designed as previously shown using the NCBI’s Primer-BLAST with a target Tm of 60°C (see Supplementary Table 1 for primer pair sequences). Thermal cycling and measurement of amplification curves were executed through the CFX Connect Real-Time PCR Detection System (Bio-Rad) and mRNA expression was calculated relative to Hprt1 and control samples as indicated, with *n* ≥ 3 biological replicates, unless otherwise noted.

## Results and Discussion

### TGFβ1-Mediated Differentiation of Liver Progenitor Cell Spheroids

To examine the broad effects of TGFβ1, which plays an important role in cholangiocytic fate, on 3D progenitor cell aggregates, we formed BMEL cell spheroids in ULA plates and AggreWells with multiple concentrations of TGFβ1 ranging from 0 to 6 ng/ml. Broadly, stem and progenitor cell differentiation is modulated by a variety of important factors such as growth factors and cell-cell interactions. At the beginning of the culture, BMEL cells were subjected to different concentrations of TGFβ1 in order to assess the effects of the latter on self-organization and differentiation of the cells.

**FIGURE 1:**
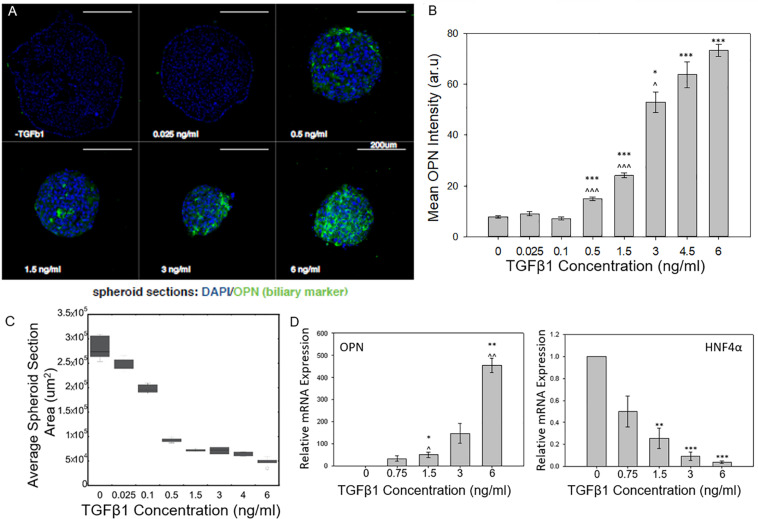
TGFβ1-mediated differentiation of liver progenitor cell spheroids. **(A)** Cross sections of BMEL spheroids immunostained for OPN with increasing TGFβ1 dosage. **(B)** Mean OPN intensity of BMEL spheroids increased as TGFβ1 concentration is increased. Student *t*-tests were performed both against 0 ng/ml TGFβ1 (*P < 0.05; ***P < 0.001) and against the next lowest TGFβ1 concentration (^*P* < 0.05; ^^^*P* < 0.001). **(C)** An increase in TGFβ1 concentration leads to a decrease in spheroid cross-sectional area **(D)** Relative mRNA expression of OPN and HNF4a against TGFβ1 concentration. (*P < 0.05; ***P < 0.001 compared to 0 ng/ml TGFβ1 and ^*P* < 0.05; ^^*P* < 0.01 compared to next lowest TGFβ1 concentration).

As the concentration of TGFβ1 was increased, the BMEL spheroids underwent increasing levels of cholangiocytic differentiation as shown by the higher expression of the biliary marker osteopontin (OPN; Figures 1A,B). The mean expression of OPN measured from the different cross sections of each spheroid observed in the ULA spheroid cross sections increased during the transition from low TGFβ1 (in the range of 0 to 0.1 ng/ml) to medium TGFβ1 concentration (0.5 to 1.5 ng/ml) and again from the medium to the high concentration (3 to 6 ng/ml). This differentiation trajectory was also seen in the smaller diameter AggreWell spheroids that showed an upregulation of OPN and a corresponding reduced expression HNF4a (a hepatocytic transcription factor) with the addition of TGFβ1 (Supplementary Figure 1). Both spheroid systems demonstrated the role of TGFβ1 in driving the 3D systems toward the cholangiocytic fate while in turn depressing the hepatocytic. Additionally, TGFβ1 treatment was observed to influence spheroid size and morphology. At higher concentrations of TGFβ1, by *t* = 72 h post-spheroid. BMEL spheroids demonstrated a more compact morphology with decreasing cross-sectional area against increasing amounts of TGFβ1 (Figures 1A,C).

Using qRT-PCR, we further examined the dose-responsive effect of TGFβ1 on spheroid differentiation. We found that an increase in OPN relative mRNA expression corresponded with increasing TGFβ1 concentration. At the highest concentration of TGFB 1 (6 ng/ml), the OPN expression was several hundred times higher than the 0 ng/ml TGFβ1 control condition. A converse relationship was observed in regards to the mRNA expression of HNF4a; in particular, treatment with increased concentrations of TGFβ1 reduced the expression of this hepatocytic differentiation marker (Figure 1D).

With this 3D spheroidal culture approach, we have confirmed that increasing the concentration of exogenous TGFβ1 leads to an enhanced induction the BMEL cell aggregates toward the biliary fate. Earlier work by Kaylan et al. (2016) measured increasing levels of TGFβ1 on BMEL cells on 2D substrates. At low doses of TGFβ1, these previous studies observed an increase in the relative expression of OPN that corresponded with an increase in TGFβ1 concentration. At what can be considered an intermediate TGFβ1 concentration (1.5 ng/ml) for *in vitro* liver progenitor experiments, these previous studies showed that the relative OPN mRNA expression exhibited a saturation behavior, and even after increasing the concentration to 6 ng/ml TGFβ1, the concentration did not increase or pass a 64 fold increase in expression compared to a 0 ng/ml TGFβ1 control. In contrasting these previous findings with the current 3D spheroid system, we found the relative expression of OPN did not saturate at intermediate TGFβ1 concentrations but continued to increase at higher doses. This was confirmed via immunostaining for OPN, which showed a continued increase in mean OPN intensity with further elevated TGFβ1 concentrations (Figure 1B). These results suggest that progenitor cells in a 3D system are more susceptible to variations in growth factor availability especially at higher concentrations compared to monolayer culture. In addition, previous studies with these progenitor cells have demonstrated that in the presence of exogenous TGFβ1, the cells undergo both morphological alterations and cellular contraction (Kaylan et al., 2016; Kourouklis et al., 2016) as they differentiate into biliary cells. Accordingly, the change in spheroid size with the addition of TGFβ1 is consistent with such cellular morphology changes. Further, our current and previous studies suggest that the *in vitro* treatment of BMEL cells with TGFβ1 can also lead to some reductions in cell numbers, likely in conjunction with the modest induction of cell apoptosis. In future work, there is potential to prevent impacts on cell survival during the biliary differentiation of progenitor cells by the replacement of TGFβ1 with alternative growth factors, or the optimization of additional co-delivered microenvironmental cues.

### Engineering Liver Differentiation Through Hydrogel Microparticles: Formulation and Uptake of Hydrogel Microparticles

**FIGURE 2:**
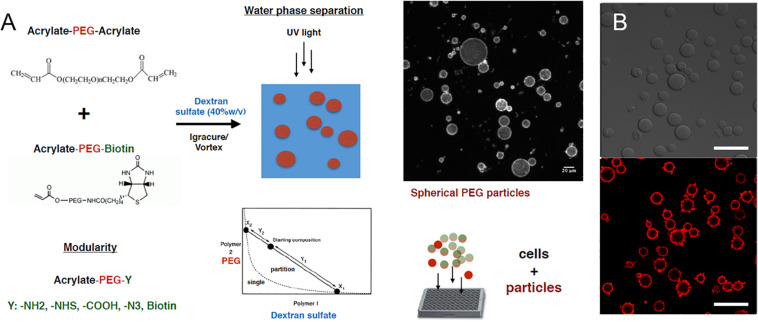
PEG hydrogel microparticle fabrication. **(A)** Fabrication of PEG hydrogel microparticles via photopolymerization of Acrylate-PEG-Acrylate with Acrylate-PEG-Biotin. **(B)** 0.7% Biotin-PEG particles with surface conjugated streptavidin-Alexa Fluor 647 as a visual marker. Scale bar: 50 μm.

**FIGURE 3:**
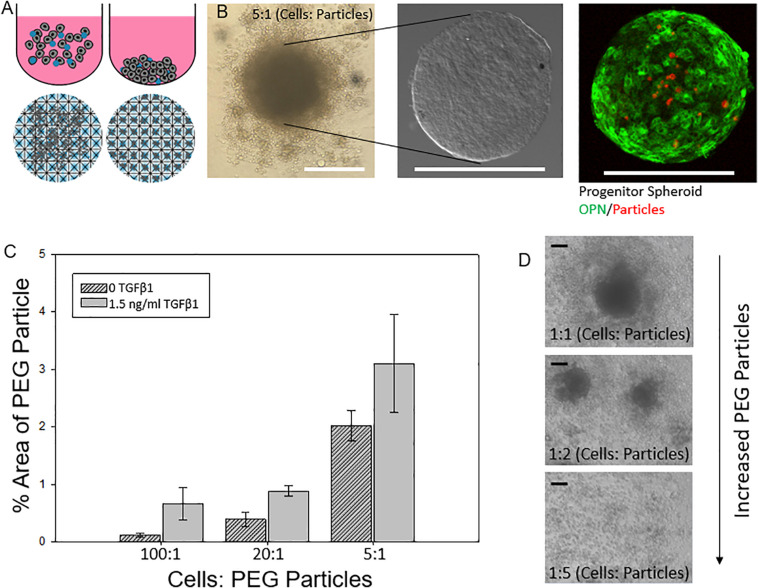
Engineering liver differentiation through hydrogel microparticles. **(A)** Spheroids made by one of two methods. **(Top)** Cells added to the wells of an Ultra Low Attachment (ULA) plate and allowed to settle and self-organize. **(Bottom)** Cells added to the wells of an AggreWell 400 plate (STEMCELL Tech, Cat.# 34421) and centrifuged to form spheroids in the microwells. Scale bars: 200 μm **(B)** ULA spheroids with PEG particles during formation- many of the PEG particles are not incorporated into the fully formed spheroid, after being removed from the well, cleaned and fixed, and after staining for differentiation markers. **(C)** Percent area of a confocal image z slice that is occupied by a PEG particle vs a cell for increasing concentrations of initial added particles with and without TGFβ1. **(D)** Integration of PEG particle into the spheroid based on initial ratio of cells: particles for spheroids with and without soluble TGFβ1. **(D)** As the relative amount of particles increases past a 2:1 cell: particle ratio, the spheroids become smaller as less cells form the initial aggregate and past a 5:1 particle to cell ratio spheroids no longer form. Scale bar: 100 μm.

We next explored the manipulation of the spheroid microenvironment via the incorporation of the PEG hydrogel microparticles. The microparticle fabrication process is described in detail in the “Materials and Methods” section and is illustrated in Figure 2. The size distribution of the hydrogel microparticles is illustrated in Supplementary Figure 2. The median microparticle radius for these studies was determined to be approximately 7 μm. We examined the effect of the relative number of microparticles within the spheroids by increasing the ratio of cells to PEG particles at the beginning of the cell seeding into the ULA plates or the AggreWell culture platform. We observed that not all of the microparticles in the initial microparticle–cell mixture got incorporated into the spheroids as the spheroidal aggregates formed (Figures 3A,B). In addition, the distribution of the microparticles throughout the spheroid was random; due to the self-organization of cells, the uptake and distribution of microparticles among the cells remained random and it is was possible to precisely control the distribution and location of the microparticles within the spheroid. The size distribution of particles within the spheroids was also random as the current and presented method of particle generation created a distribution of particle sizes in the single cell size range (Supplementary Figure 2). A future study involving monodisperse microparticles, with more tightly controlled microparticle dimensions, could potentially illustrate a microparticle integration pattern that is not observed with the diverse particles created here. Nonetheless, we observed that final number of microparticles was increased in accordance with their differences in the initial seeding density and independent of the treatment with TGFβ1. Specifically, this increase in microparticle incorporation due to an increase in relative microparticle numbers was observed both for spheroids formed with and without the presence of TGFβ1 (Figure 3C). There was a modest increase in the number of microparticles incorporated into spheroids treated TGFβ1, which may have been a consequence of the enhanced compaction of TGFβ1-treated spheroids relative to untreated controls (Figure 1). Through manipulation of the cell to microparticle ratio, we also found that increasing the number of PEG microparticles interfered with spheroid formation; specifically, at a 1:1 cell to microparticle ratio, the resulting spheroids were smaller compared to lower particle ratios leaving a significant number of cells outside of the forming spheroid. At 1:2 cell to microparticles, there was no longer a single spheroid and distinct, and smaller spheroids were formed. At 1:5, there was no longer any spheroid formation (Figure 3D), as the PEG microparticles inhibited the aggregation of the progenitor cells at this ratio.

### Hydrogel Microparticles Influence Progenitor Cell Differentiation in a TGFβ1-Dependent Manner

**FIGURE 4:**
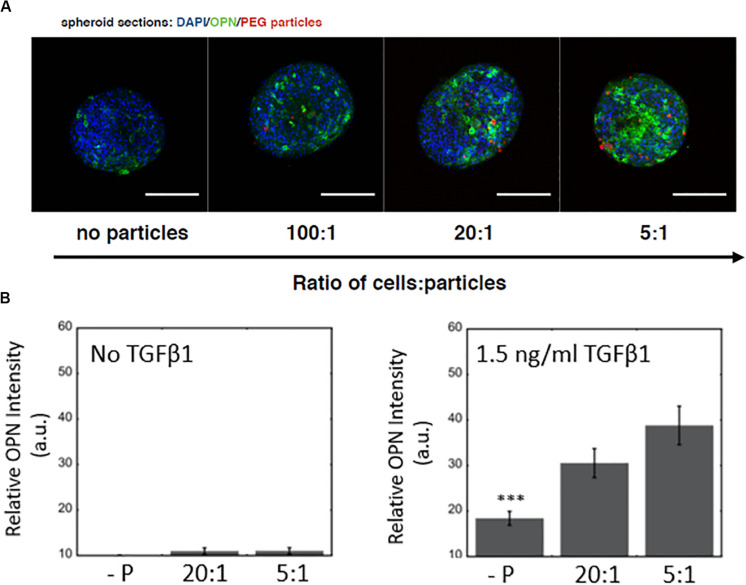
Microparticle integration at a constant TGFβ1 dose and differentiation response for spheroids embedded with PEG hydrogel microparticles **(A)** Increased OPN expression with an increase in PEG microparticle concentration. No particle controls (-P). Scale bars: 150 μm. **(B)** Without TGFβ1, increasing microparticle density does not have any significant effect on OPN expression, while at a constant TGFβ1 concentration (1.5 ng/ml), there is a difference between spheroids with and without microparticles.

Next, we examined the effect of microparticle density on the differentiation of BMEL cells following microparticle integration into the spheroidal aggregate cultures. In these studies, the hydrogel microparticles were encased by the 3D cell clusters during formation and remained within the spheroids throughout the maturation of the cultures. We showed that an increased number of hydrogel microparticles relative to BMEL cells was sufficient to incorporate an increased number of microparticles within the differentiating spheroids (Figure 4A). In addition, exogenous treatment with the growth factor TGFβ1, together with the introduction of the microparticles, exhibited a synergistic effect on differentiation. Specifically, spheroids with an increased number of microparticles demonstrated an enhancement of biliary differentiation at the same concentration of TGFβ1, relative to the spheroidal aggregates formed without the PEG microparticles (Figure 4B). These results suggest that the addition of PEG hydrogel microparticles, without any additional active surface chemistry, was sufficient to modulate the differentiation trajectory. Possible mechanisms underlying this effect include the influence of integrated microparticles on the collective biomechanical stiffness of the spheroid, or the potential modulation of the diffusive characteristics of the spheroid in response to microparticle incorporation, therefore, enabling greater growth factor penetration. As previously discussed, these progenitor cells undergo a morphological change and contract in the presence of TGFβ1. Integrated particles could potentially modulate this contraction process. It is also possible that there is an underlying effect of the streptavidin used to coat the microparticles for subsequent labeling with fluorescent tags and other moieties. Previous work has demonstrated that RYDS motifs on streptavidin have been shown to bind cell surface integrins (Murray et al., 2002). Therefore, it is possible that the presence of streptavidin could support some adhesion-based signaling within the aggregate. Future studies utilizing a different variant of streptavidin, such as Neutravidin, or an overall distinct attachment scheme, may be used to investigate this possibility and any potential role for integrin engagement. Additionally, future efforts could be undertaken to modify the surface of these microparticles, with charged or other adhesive groups, to further enhance spheroidal integration. However, any alterations that lead to biochemical or mechanical interactions within the spheroidal culture could produce secondary effects on differentiation trajectories, that would need to be evaluated in parallel.

### Adding Surface Conjugated Heparin to Microparticles to Tune Liver Differentiation

**FIGURE 5:**
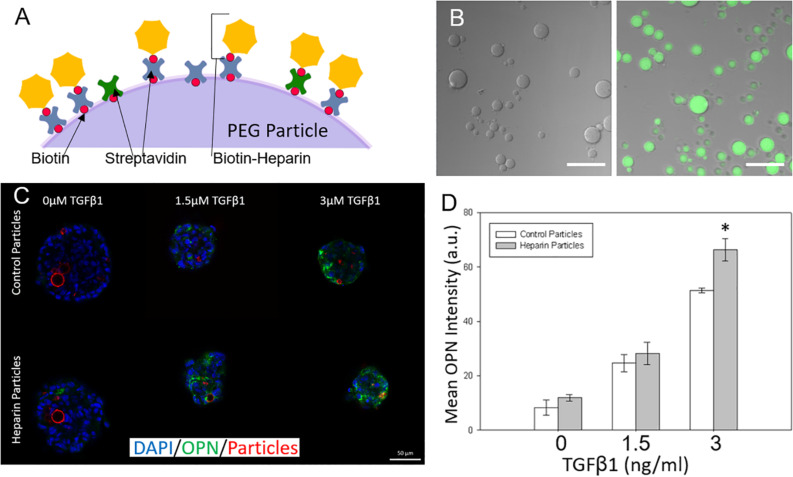
Adding surface conjugated heparin to microparticles to tune liver differentiation. **(A)** Biotinylated heparin is conjugated to the PEG particle surface via a biotin-streptavidin-biotin bridge. **(B)** Heparin presence on PEG particles was verified by conjugating AF488 fluorescent heparin to particles and imaging. **(Left)** control particle without heparin addition. **(Right)** AF488-heparin conjugated to particle. Scale Bars: 50 μm **(C)** Heparin and control particles integrated into Aggrewell spheroids and exposed to different levels of TGFβ1 (Blue: DAPI; Green; OPN; and Red: AF647-PEG particles) **(D)** Mean OPN intensity for Aggrewell spheroids with the 2 particle types.

After examining the effects of unmodified hydrogel microparticles, we sought to investigate if the surface functionalization of the microparticles could further influence the differentiation process (Benoit et al., 2008; Lutolf et al., 2009). In particular, we conjugated the glycosaminoglycan heparin to the PEG microparticles due to its known ability to bind and sequester growth factors (Rinker et al., 2018). This was achieved by the addition of biotinylated heparin to the microparticles via the incorporation of acrylate-PEG-biotin into the PEG microparticles during fabrication, and the post-particle fabrication treatment with streptavidin, to establish a streptavidin bridge (Figures 5A,B). Heparin conjugation was verified by using Alexa Fluor 488-labeled heparin-biotin, and the assessment of microparticle fluorescence using microscopy (Figure 5B).

### Modifying Particles With Heparin Alters Progenitor Differentiation

Differentiation studies demonstrated that spheroids with induced heparin-conjugated PEG microparticles expressed higher OPN than spheroids with unmodified microparticles, particularly at higher TGFβ1 concentrations (Figures 5C,D). At higher concentrations of TGFβ1 (3 ng/ml), smaller Aggrewell-formed spheroids with heparin particles expressed OPN at ∼1.25 fold over the spheroids with standard particles. Spheroids were further treated with SB-43154 (a TGFβ1 inhibitor) to show the continued importance, and specificity, of the effect of the growth factor TGFβ1 on differentiation within the combined growth factor and heparin coated particle system by eliminating the biliary differentiation (Supplementary Figure 3).

The effects of heparin-conjugated microparticles on differentiation behavior were mirrored in the analysis of OPN mRNA expression, using mRNA isolated from progenitor cells cultured within the larger ULA spheroids. In three separate experiments, we demonstrated that at the relatively high TGFβ1 concentrations, there was increased OPN expression in spheroids containing heparin-modified microparticles compared to those containing unmodified PEG microparticles (Supplementary Figure 4). In each replicate experiment, spheroids exposed to high concentrations of TGFβ1 (3 and 6 ng/ml) showed increased expression with statistically significant (*P* < 0.5) increases at the highest concentration (6 ng/ml). Although there was variation in the expression of OPN relative to the undifferentiated progenitor cells, the effect of heparin-conjugated microparticles on the relative enhancement of OPN expression at higher TGFβ1 concentrations, was consistently observed across replicate studies.

Overall, modifying the surface of our integrated PEG microparticles with heparin shifted the differentiation trajectory of the spheroids with increasing levels of TGFβ1. Heparin has been demonstrated to bind a broad range of growth factors, and our results demonstrate that at higher concentrations of TGFβ1 there was an increase in the expression of a marker of biliary differentiation that has been previously established to be induced by TGFβ1 treatment. Consequently, we hypothesize that the heparin presented by the modified PEG microparticles may sequester TGFβ1 in the spheroid, and thereby facilitate an increased local concentration of TGFβ1, which subsequently leads to an enhancement of biliary differentiation (Rinker et al., 2018). In future efforts, to mitigate some of the variations observed across distinct microparticle culture experiments, a change in the particle formation to select for more narrow range of particle sizes, or the incorporation of defined microparticle surface modification that could enable a better standardization in regards to microparticle spheroidal integration, may help to control these variations. In addition, complementary studies incorporating techniques such as fluorescence resonance energy transfer (FRET) to directly evaluate the effect of microparticle-presented heparin on the molecular interaction with growth factors such as TGFβ1 within the spheroid, could provide insights into the mechanisms underlying the differentiation effects and provide a blueprint for further tuning the microparticle approach.

## Conclusion

In conclusion, we have established a 3D culture system to study and subsequently modulate liver progenitor cell differentiation based on the concentration of TGFβ1 and the incorporation of cell-sized hydrogel microparticles. Liver progenitor cell fate was dependent on the concentration of the exogenous growth factor, but by modulating the amounts and surface chemistry of PEG microparticles within the spheroids, we demonstrated the potential to further tune this differentiation process, including a specific enhancement of liver progenitor differentiation toward the biliary lineage. Overall, these efforts demonstrate the utility of a microparticle integration approach for the systematic study of progenitor cell differentiation and represent an important building block toward the improved understanding of the nature of bipotential liver progenitor cells in the context of complex 3D microenvironments.

## Data Availability Statement

All datasets generated for this study are included in the article/Supplementary Material.

## Author Contributions

SG: overall experimental design and execution, data collection and analysis, and text and figure preparation. AK: experimental design and execution, data collection and analysis, and text and figure preparation. HR: experimental implementation and data collection and analysis (confocal imaging and PCR). GU: text and figure preparation. All authors contributed to the article and approved the submitted version.

## Conflict of Interest

The authors declare that the research was conducted in the absence of any commercial or financial relationships that could be construed as a potential conflict of interest.
