# Supplementary material for: Integration of Hydrogel Microparticles With Three-Dimensional Liver Progenitor Cell Spheroids
Source: Front Bioeng Biotechnol. 2020 Jul 21;8:792. doi: 10.3389/fbioe.2020.00792 (PMC7385057; doi:10.3389/fbioe.2020.00792)
Supplement: Supplementary file 1 [file Data_Sheet_1.docx]

**Supplemental Material**

**Integration of hydrogel microparticles with three-dimensional liver progenitor cell spheroids**


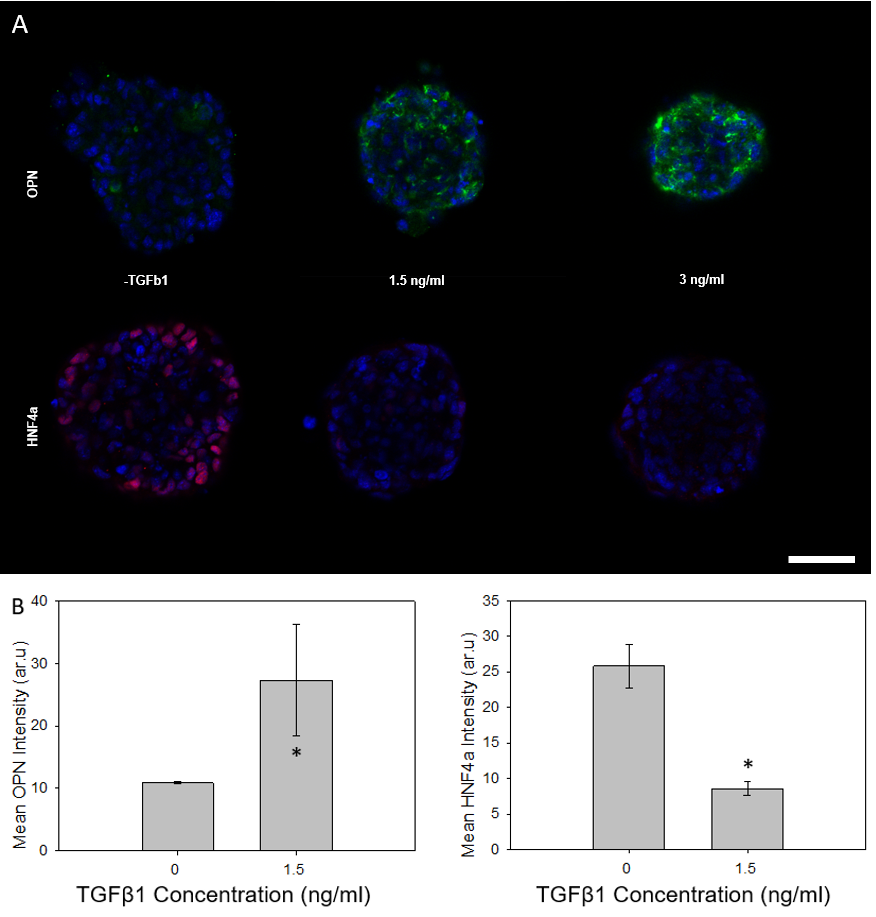


**Supplemental Figure 1:** (A) AggreWell spheroids stained for OPN (Green) and HNF4a (Red) at 0, 1.5 and 3 ng/ml TGFβ1, Scale bar: 50 µm (B-C) Relative fluorescent intensity and expression of OPN and HNF4a for AggreWell spheroids. *: P < 0.05

**
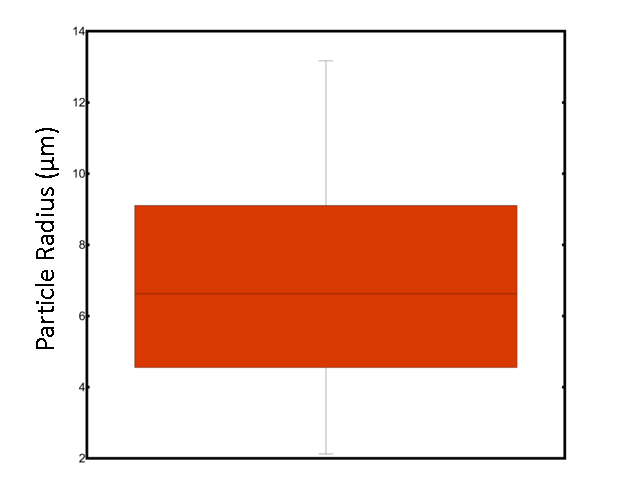
**

**Supplemental Figure 2:** Hydrogel microparticle size distribution. Median microparticle radius for these studies was determined to be approximately 7 µm.


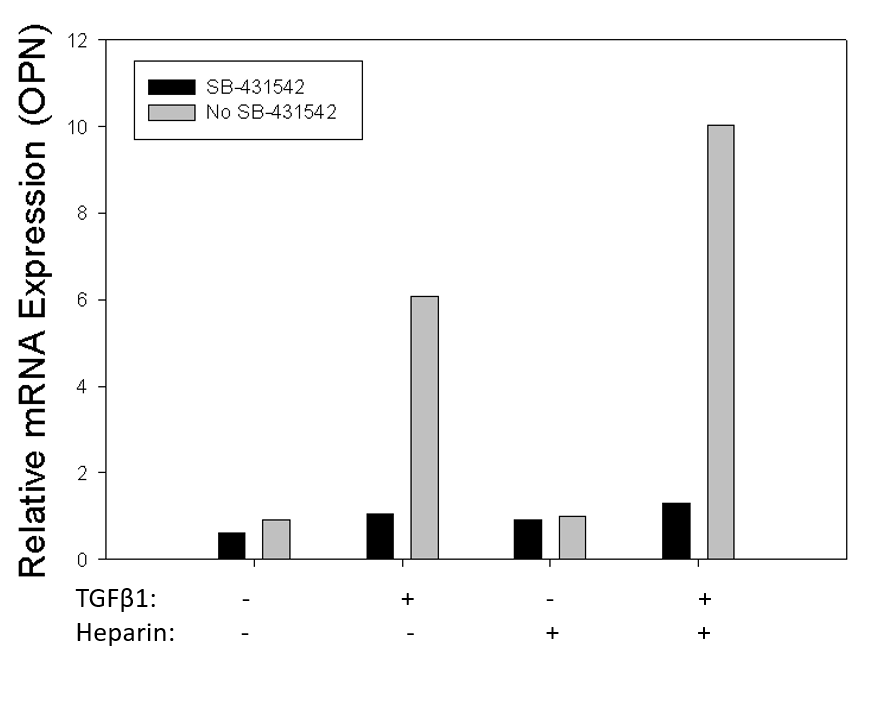


**Supplemental Figure 3:** Relative OPN mRNA expression for spheroids treated with the SB-43154 TGFβ (type I) receptor inhibitor, in the presence or absence of TGFβ1 treatment (1.5 ng/mL).


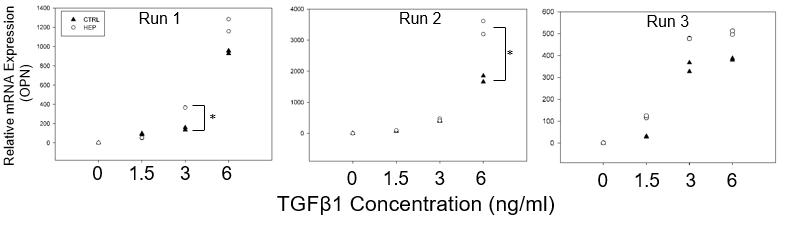


**Supplemental Figure 4:** Three distinct qRT-PCR replicate experiments demonstrated that heparin-conjugated PEG microparticles promote increased OPN mRNA expression in spheroids in a TGFβ1 concentration dependent manner.

**Supplemental Table 1**: PCR primer pairs

| **Gene** | **Sequence (5’ to 3’)** |
| --- | --- |
| Hprt1 | Forward: GGAGTCCTGTTGATGTTGCCAGTA  Reverse: GGGACGCAGCAACTGACATTTCTA |
| OPN | Forward: ACTACGACCATGAGATTGGCAGTG  Reverse: CTATAGGATCTGGGTGCAGGCTGTAAA |
| HNF4α | Forward: ATGCGACTCTCTAAAACCCTTGCC  Reverse: CCAGGCTGTTGGATGAATTGAGGT |
